# Efficacy and Safety of intravenous monoclonal antibodies in patients with moderate-to-severe active Graves’ophthalmopathy: a systematic review and meta-analysis

**DOI:** 10.3389/fendo.2023.1160936

**Published:** 2023-05-23

**Authors:** Yu Hu, Jinhua Chen, Ken Lin, Xijie Yu

**Affiliations:** ^1^ Laboratory of Endocrinology and Metabolism/Department of Endocrinology and Metabolism, Rare Disease Center, West China Hospital, Sichuan University, Chengdu, China; ^2^ Department of Endocrinology and Metabolism, Chengdu First People’s Hospital, Chengdu, China; ^3^ Department of General Practice, Chengdu First People’s Hospital, Chengdu, China

**Keywords:** monoclonal antibodies, tocilizumab, teprotumumab, rituximab, Graves ophthalmopathy, treatment, meta-analysis

## Abstract

**Backgrounds:**

The effects of various treatments on Graves’ ophthalmopathy (GO) have been studied. As monoclonal antibodies (mAbs) have been proposed for the treatment of moderate to severe GO, direct comparisons between different mAbs are lacking.We therefore conducted this meta-analysis to objectively compare the efficacy and safety of intravenous mAbs.

**Methods:**

To identify eligible trials, references published before September 2022 were electronically searched in PubMed, Web of Science, Pubmed, Embase,Cochrane Library, CBM, CNKI,Wan-Fang and ICTRP databases.The Newcastle-Ottawa scale (NOS) and the Cochrane Risk of Bias Assessment Tool were used to assess the risk of bias of the original studies.The primary and secondary outcomes were the response and inactivation rates, with the secondary outcomes being the clinical activity score (CAS),the improvement of proptosis and diplopia improvement,and the adverse event rate. Publication bias was evaluated, along with subgroup and sensitivity analyses.

**Results:**

A total of 12 trials with 448 patients were included. The meta-analysis showed that TCZ (tocilizumab) was most likely to be the best treatment in terms of response according to indirect contrast, followed by TMB (teprotumumab) and RTX (rituximab).TCZ, followed by TMB and RTX, was also most likely to be the best treatment in terms of reducing proptosis. In terms of improving diplopia, TMB was most likely to be the best treatment, followed by TCZ and RTX.TCZ was the highest probability of safety, followed by RTX and TMB.

**Conclusions:**

Based on the best available evidence,TCZ should be the preferred treatment for moderate to severe GO.In the absence of head-to-head trials,indirect comparisons of treatments are routinely used to estimate the effectiveness of the treatments of interest. In addition,the optimal dose and potential mechanism of action of monoclonal antibodies remain to be established,and it is encouraging that the treatment paradigm for GO may change in the future.

This study was designed in accordance with the Preferred Reporting Items for conducting Systematic Reviews and Meta-Analyses (PRISMA)(27).

**Systematic Review Registration:**

http://www.crd.york.ac.uk/prospero, identifier CRD42023398170.

## 1 Introduction

Graves’ ophthalmopathy (GO) is a complex autoimmune disease of the orbit caused by progressive inflammation and damage to the orbital and ocular tissues (1, 2). It is the most important and typical extrathyroidal manifestation of Graves’ disease (3) and causes enlargement of the retro-orbital fat and extraocular muscles, thought to be mediated primarily by upregulation of the insulin-like growth factor 1 receptor on orbital fibroblasts (1). The prevalence of GO ranges from 0.1% to 0.3% (4) and is sight-threatening in 3-5% of patients and clinically relevant in 25-50% of patients with Graves’ disease (5). It can cause ocular symptoms such as periorbital oedema and chemosis, lid retraction, diplopia, proptosis, exposure keratopathy and dysthyroid optic neuropathy (DON). Severe proptosis can lead to disfiguring facial changes, disabling diplopia and, in severe cases, visual impairment (6–8) and may occur before, after or concurrently with Graves’ disease. These symptoms have a variable impact on patients’ quality of life (9–12). In fact, two main processes are involved in GO, namely cellular and humoral immunity. T lymphocytes, to which antigen-presenting cells and B lymphocytes present anti-TSH receptor antibodies, are involved in cellular immunity. Activation of B lymphocytes results in the secretion of various cytokines, including tumour necrosis factor alpha (TNF-α), interferon gamma (IFN-γ), interleukin-1 (IL-1) and interleukin-6 (IL-6), which primarily target the orbital adipocyte, inducing its differentiation into a mature adipocyte and the synthesis of glycosaminoglycans (GAGs), particularly hyaluronan (HA). This results in orbital muscle and connective tissue oedema, orbital fat hypertrophy and signs of inflammation, making it crucial to find a safe and effective treatment for GO (13–15), the pathogenesis of which remains poorly understood and the treatment of which is controversial (7).

Depending on the activity and severity of GO, medications, radiotherapy and eye surgery have been used to improve symptoms. The European Group on Graves’ Orbitopathy (EUGOGO) has reached a consensus that all but the mildest patients with GO should be referred to multidisciplinary clinicians for further evaluation and management, and that intravenous glucocorticoids (GCs) are used to treat active ophthalmopathy.Surgical decompression is considered in the stable phase or in an emergency (sight-threatening or corneal collapse) (16). Thus, GCs have been the mainstay of treatment for the past six decades, with oral, intravenous or topical injections being the most common and widely used immunosuppressive agents for active and moderate to severe GO (17–19), as further recommended by EUGOGO (14). The treatment of GO remains challenging and often unsatisfactory, although several approaches have been used (20). To prevent the progression of the autoimmune disease, GCs play a beneficial role in reducing inflammation and congestion in the orbital tissues.However, high doses of GCs are usually associated with adverse events, such as glycaemia, cushingoid features,weight gain,liver damage,peptic ulcer, and cardiovascular complications (21, 22). Morbidity and mortality were reported to be 6.5% and 0.6%, respectively, in patients undergoing intravenous GC therapy for GO (23). In addition, the non-response rate was approximately 20-25% and a further 10-20% of patients experienced disease relapse after discontinuation of GCs (24, 25). In the event of a lack of response, partial response or adverse reactions to first-line treatment, there is an urgent need for a rapid switch to an effective second-line therapy. Therefore, alternative treatment modalities are urgently needed.

In recent years, with advances in the pathophysiology of GO and monoclonal antibody technology, immunotherapy targeting different molecular pathways from Thyroid Stimulating Hormone Receptor (TSHR) to insulin-like growth factor 1 receptor (IGF-1R), from cytokine mechanisms such as TNF-alpha and IL-6R to Tregs and beyond has been investigated and shown to be a promising therapeutic alternative or adjunct to treatment (26). More recently, TCZ (targeting the IL-6 receptor), RTX (targeting CD20), TMB (a human anti-IGF-1R monoclonal antibody) have been shown to be effective and safe therapeutic options in the treatment of refractory GO. Effective early treatment of these patients is predictive of a favorable outcome (8) and reduces the need for surgery for second-stage disease sequelae. Therefore, the present systematic review aimed to investigate the relative efficacy and safety of intravenous monoclonal antibodies as a therapy for GO, as to our knowledge there are currently no comparative studies on the efficacy of monoclonal antibodies.

## 2 Methods

### 2.1 Protocol and registration

This study was designed in accordance with the Preferred Reporting Items for conducting Systematic Reviews and Meta-Analyses (PRISMA) (27). Consent for this analysis was registered with PROSPERO (http://www.crd.york.ac.uk/prospero), registration number CRD42023398170.

### 2.2 Data sources and search strategy

A comprehensive search strategy was used to identify relevant English-language literature in the following electronic databases: Web of Science, Pubmed, Embase, Cochrane Library, Chinese Biomedical Literature Database (CBM), China National Knowledge Internet (CNKI), Wan-Fang digital database and WHO International Clinical Trial Registration Platform (ICTRP). A manual search was performed when necessary. The electronic search covered the period from April 1966 to September 2022. The following search terms were used: “monoclonal antibody” or “rituximab” or “rituxan” or “teprotumumab” or “etanercept” or “K1-70” or “adalimumab”, and “endocrine ophthalmopathy” or “Graves’ ophthalmopathy” or “dysthyroid ophthalmopathy” or “thyroid ophthalmopathy” or “thyroid-associated ophthalmopathy” or “Graves’ orbitopathy” or “endocrine orbitopathy” or “thyroid orbitopathy” or “Graves’ eye disease” or “thyroid eye disease”. We also screened the reference lists of all included trials, relevant systematic reviews and previous meta-analyses to identify additional trials not included in the primary search.

### 2.3 Selection and eligibility criteria

Two reviewers (YH, JHC) independently screened references and abstracts retrieved from the primary search for eligible studies. Discussion and a third reviewer (XJY) were used to resolve disagreements.This meta-analysis included published studies that met the following selection criteria:(i) study design (i.e. randomised controlled trial or cohort study including intravenous monoclonal antibody therapy, excluding other combination interventions for the treatment of GO);(ii) population (iii) intervention (i.e. monoclonal antibody or placebo); (iv) outcome variables (i.e. at least one of the following outcome variables: disease response rate, disease inactivation rate, CAS, proptosis and diplopia). Exclusion criteria were the following:(i) reviews, systematic reviews or meta-analyses, commentaries, letters, case reports, conference abstracts or *in vitro* studies;(ii) failure to meet the above-mentioned GO diagnostic criteria;(iii) failure to strictly follow the advice of the physician during the procedure or loss to follow-up during the procedure or acceptance of other special treatments that affect the observation indicators of this study;(iv) studies with insufficient data to extract or calculate results; and (v) studies with duplication of data or repeated analyses.

### 2.4 Data extraction and results

#### 2.4.1 Data extraction

Data from the trials were extracted using a specially adapted form and then jointly reviewed by two independent reviewers (YH, JHC). The following information was extracted and databased:title, sample size, inclusion/exclusion criteria, baseline characteristics of participants,interventions, outcome measures,adverse events and follow-up. We estimated data from graphs using Plot Digitizer (version 2.6.8) when exact data were not available in the article.

#### 2.4.2 Quality assessment

For randomized controlled trials (RCTs), the Cochrane Collaboration Risk of Bias tool was used to independently assess the methodological quality of eligible trials by two reviewers (28). Each term had three levels of difficulty on the basis of seven aspects, one of which was the generation of random sequences, allocation concealment, blinding of participants and personnel, blinding of outcome assessment, integrity of the outcome data, selective reporting, and other biases. Risk of bias graphs were generated using RevMan5.3 software.For non-RCTs, two reviewers rated all included studies using the NOS, which consists of study group selection, comparability and exposure (29). The total score ranged from 5 to 9, with a higher score representing a higher quality assessment, and studies with ≥7 score were considered to be of high quality. Consensus discussion with a third author resolved any discrepancies or low levels of agreement.

#### 2.4.3 Outcome measures

The primary outcome measure was each trials defined response rate (i.e. the ratio of responders to total patients),and the secondary outcome measures were disease inactivation rate, reduction in CAS, reduction in proptosis, and improvement in diplopia from baseline to the end of follow-up. Tolerability was assessed by calculating the proportion of patients with adverse events in each regimen. Where a response rate figure was available, it was used directly. Where these were not available, we used the following criteria (in the order given): CAS reduction ≥2, ophthalmoscopic index reduction ≥2, proptosis reduction ≥2 mm and no need for additional therapy.

### 2.5 Statistical analysis and quality assessment

#### 2.5.1 Statistical analysis

R (version 4.2.0) was used for statistical analysis, and the Freeman-Tukey double inverse sine transformation was used to transform data for dichotomous variables that did not follow a normal distribution, otherwise the original data were used as effect sizes. Cochran’s Q statistic and I^2^ values were used to assess heterogeneity between studies. I^2^ describes the percentage of the total change that is caused by heterogeneity between studies and not by chance. The random effects model with 95% CI was used as the combined method when heterogeneity was high (I^2^ >50%).The discussion of possible sources of heterogeneity mainly used subgroup analysis (by study design type: RCT and non-RCT), and sensitivity analysis (one by one method) was used to screen the studies with a large effect on heterogeneity. Funnel plots were used to assess publication bias when more than 10 studies were included, and sensitivity analysis was used to assess the stability of the results. The symmetry of the funnel plot was used to assess publication bias, and the Peters test (or Egger test) was applied, including the Peters test for bicategorical variables and the Egger test for continuous variables. All tests were two-tailed and p<0.05 was considered statistically significant.

## 3 Results

### 3.1 Identification and selection of studies

**Figure 1:**
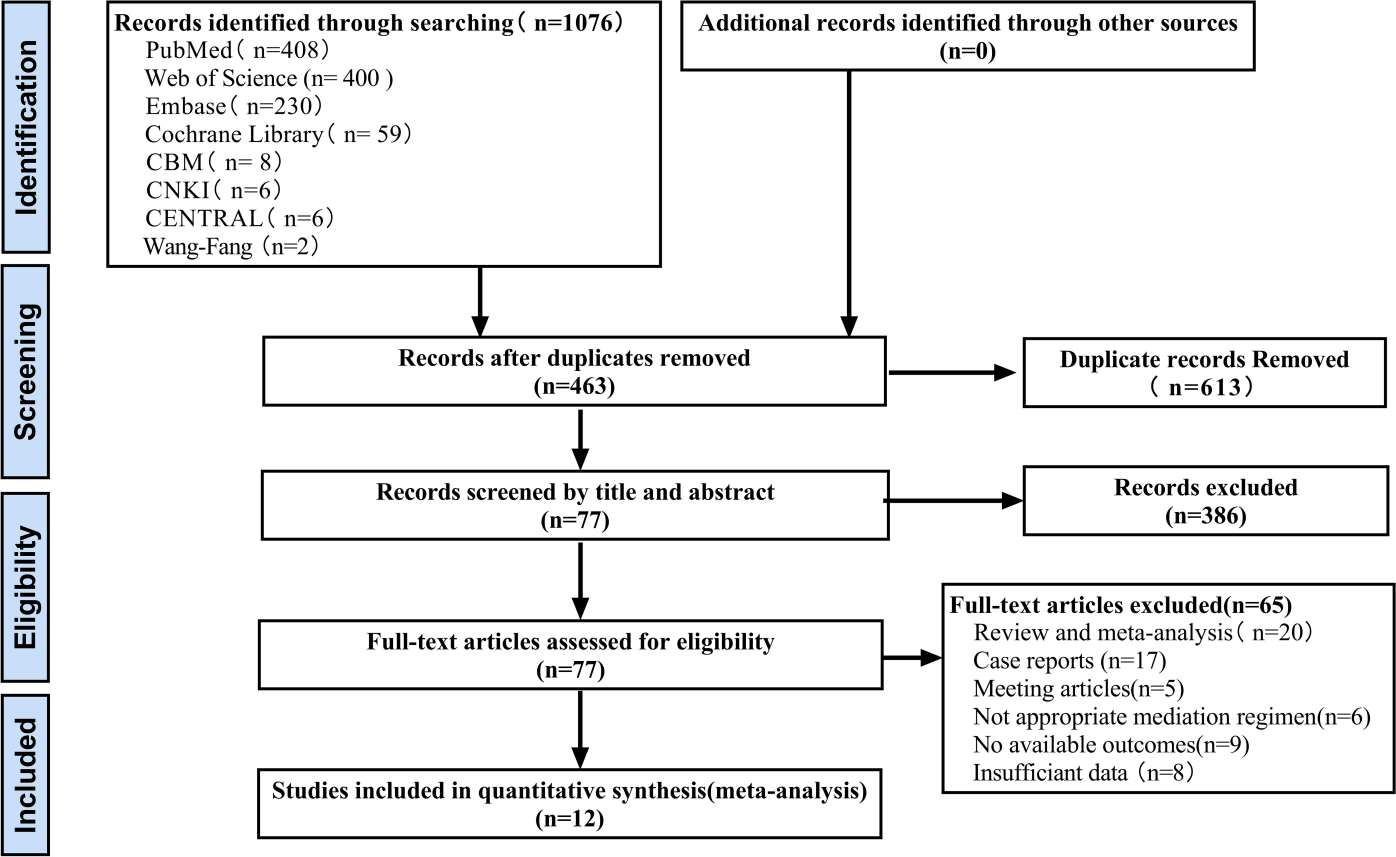
The process of identifying trials eligible for inclusion in the meta-analysis.

A total of 1076 records were initially identified after electronic searches of PubMed (n=408), Web of Science (n=400), Embase (n=230), Cochrane Library (n=59), CBM (n=8), CNKI (n=6), Central Register of Clinical Trials (CENTRAL) (n=6) and Wanfang digital database (n=2). All records were downloaded and imported into EndNote X9.A total of 613 duplicates were removed using the duplicate detection function. After excluding ineligible records (n=386) based on title and abstract screening, full text articles were assessed (n=65), a total of 12 eligible studies were included in the final meta-analysis. The process of identifying and selecting eligible studies is shown in **Figure 1**.

### 3.2 Included eligible study characteristics

All eligible studies had a publication date between 2001 and 2021.Of the 12 eligible studies, the sample size of each study ranged from 33 to 90, with a median sample size of 57 and a cumulative sample size of 593.12 trials enrolled patients with moderate-to-severe active GO, of which 5 trials were RCTs and 7 trials were observational studies. The baseline characteristics details of the 12 eligible trials are shown in **Table 1**.

**Table 1:** Characteristics of included studies in the meta-analysis.

Study(author/year)	Study Characteristics						Baseline Characteristics						
	Baseline simple size T(C)	Location	Treatments group (Control)	Study design	Single vs Multicenter	Main Outcomes	Mean Age,y	Female NO. (%)	CAS mean (SD)/ median (IQR)	Proptosis,mm mean (SD)/ median (IQR)	Diptopia NO. (%)	Follow-up	Study quality
**Salvi 2015** (30)	15 (17)	Italy	RTX(IVGC)	RCT	**S**ingle	CAS	51.9	14 (93.3)	4.4 (0.7)	OS:23.5 (3.5) OD:23.2 (2.5)	9 (66.7)	52wks	–
**Stan 2015** (26)	13 (12)	USA	RTX (placo)	RCT	**S**ingle	CAS	57.6	9 (69.2)	4.9 (1.0)	OS:24.2 (3.3) OD:24.6 (3.0)	NR	52wks	–
**Deltour 2020** (31)	23	France	RTX(pre-post)	RC	Multicentric	CAS	51.2	15 (67)	4.09 (0.79)	21.84 (2.59)	NR	24wks	NOS: 6
**Eid 2019** (32)	14	France	RTX(pre-post)	RC	**S**ingle	CAS	60	9 (60)	4.0 (3.0-4.0)	NR	NR	24wks	NOS: 5
**Perez-Moreiras 2018** (33)	15 (17)	Spain	TCZ(placo)	RCT	Multicenter	CAS	45.07	11 (73.3)	5 (5.0-7.0)	21 (19.5-23)	NR	40wks	–
**Pérez-Moreiras 2021 (** 34)	54	Spain	TCZ(pre-post)	RC	**S**ingle	CAS TRAb levels	53.8	41 (75.9)	6.7 (1.5)	21.8 (15–29)	40 (85.1)	16wks	NOS: 7
**Sánchez-Bilbao 2020 (** 35)	45	Spain	TCZ(pre-post)	RC	Multicenter	BCVA, CAS, IOP	51	38 (79.2)	4.64 (1.5)	NR	NR	48wks	NOS: 6
**Pérez-Moreiras 2014 (** 36)	18	Spain	TCZ(pre-post)	PC	**S**ingle	CAS	47.94	16 (88.9)	6.5 (1.29)	22.33 (3.16)	7 (38.9)	36wks	NOS: 7
**Smith 2017 (** 37)	42 (45)	USA	TMB(placo)	RCT	Multicenter	CAS Proptosis response	51.6	28 (65)	5.1 (0.97)	23.4 (3.2)	38 (90)	24wks	–
**Douglas 2020 (** 38)	41 (42)	USA&Europe	TMB(placo)	RCT	Multicenter	CAS Proptosis response	51.6	29 (71)	5.1 (0.9)	22.62 (3.32)	NR	24wks	–
**Douglas 2021 (** 39)	14	USA&Europe	TMB(pre-post)	PC	Multicenter	Proptosis response	56.1	11 (78.6)	3.6 (1.7)	23.0 (3.1)	NR	28wks	NOS: 8
**Bennedjaï 2020 (** 40)	21 (7)	France	RTX (TCZ)	RC	Multicenter	CAS	50.0	14 (66.7)	5(0.5)	NR	NR	44wks	NOS: 8

RTX, rituximab; TCZ,tocilizumab; TMB,teprotumumab; IVGC, intravenous glucocorticoids; RCT, randomized controlled trial; RC, retrospective cohort; PC, prospective cohort; CAS, clinical activity score; NR, not reported.

### 3.3 Risk of bias

**Figure 2:**
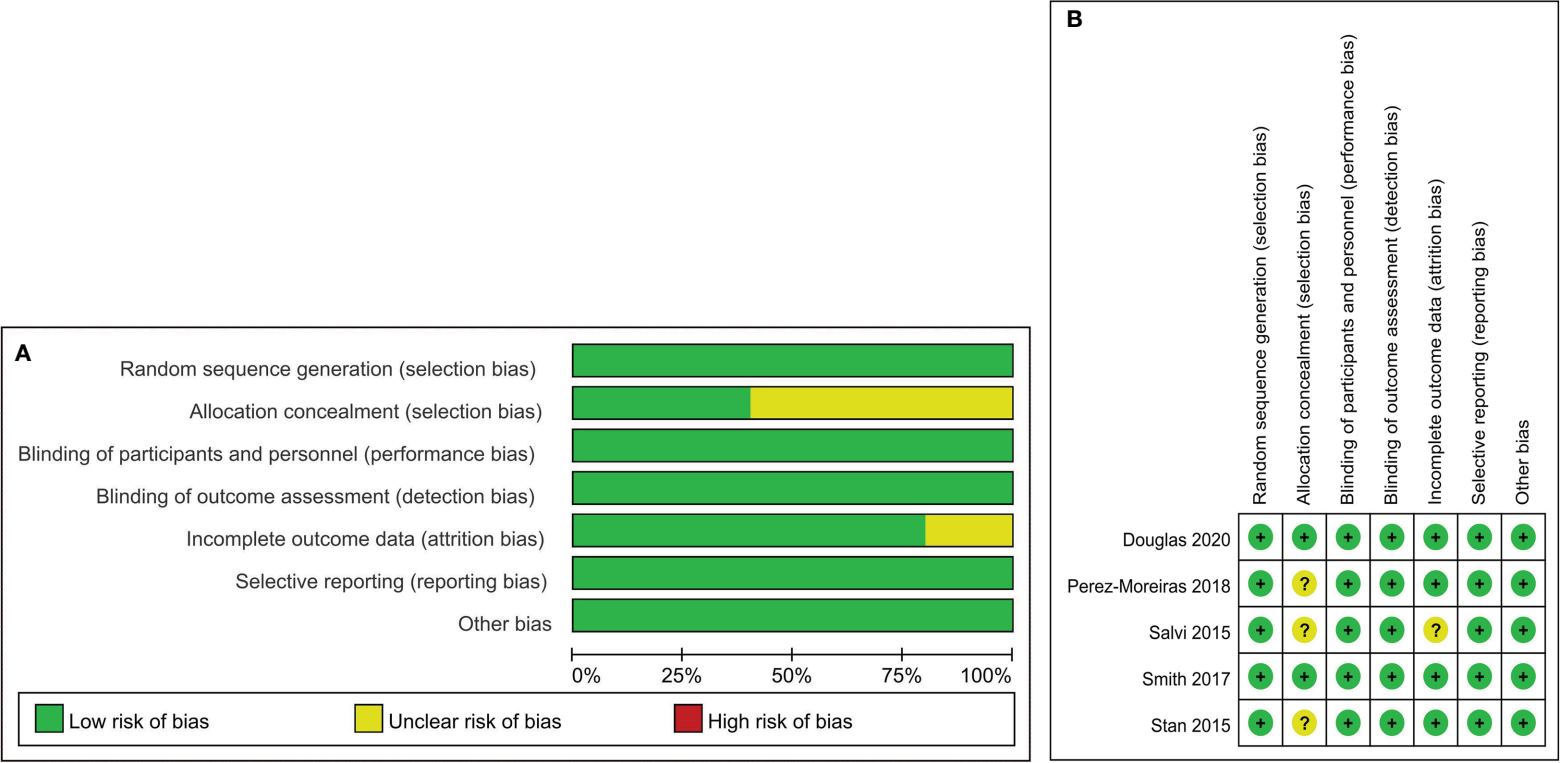
Risk of bias summary and Risk of bias graph. Risk of bias summary **(A)**: a review of authors’ judgments about each risk of bias item for each included study. Risk of bias graph **(B)**: A review of authors’ judgments about each risk of bias item presented as percentages across all included studies.

In this meta-analysis, the risk of bias of 5 RCTs was assessed using the Cochrane Risk of Bias Tool.A total of 5 trials reported details of the of random sequence generation, blinding of participants and personnel, outcome assessment, and selective reporting. 2 trials clearly described the methods used to conceal allocation.The details of assessing risk of bias are summarised (**Figure 2**). For 7 observational trials, 4 trials with NOS scores were considered to be of high quality (**Table 1**). 12 studies with complete data or use of appropriate statistical methods, and all studies reported expected outcomes. Funnel plots were used to assess publication bias.

### 3.4 Effectiveness

#### 3.4.1 Response rate

**Figure 3:**
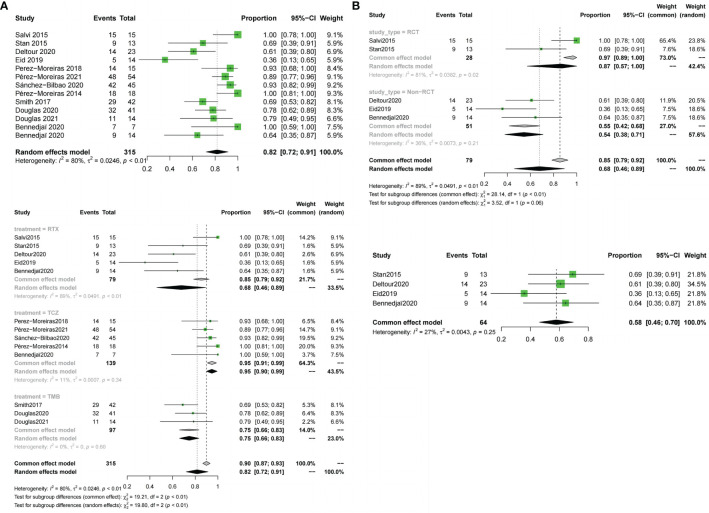
Forest plot and Funnel plots of response rate. **(A)** Forest plot of response rate (Combined analysis and Drugs subgroup). **(B)** Forest plot of response rate (RTX subgroup).

**Figure 4:**
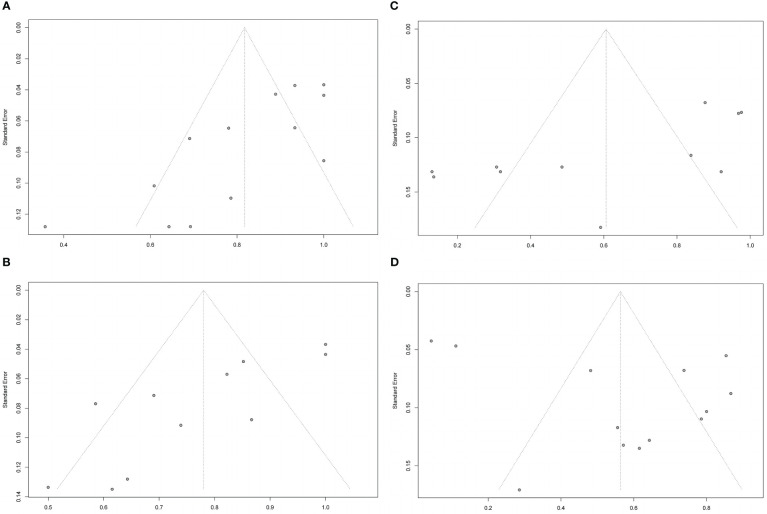
Funnel plots of response rate,disease inactivation rate, improvement in diplopia and adverse events rate. Funnel plots of response rate **(A)**, disease inactivation rate **(B)**, improvement in diplopia **(C)** and adverse events rate **(D)**.

All 12 included trials reported the response rate.The meta-analysis concluded that all the trials of the three drugs,obtained the combined value of the response rate (OR:0.82;95%CI=0.72-0.91,random effect model,I^2^ = 80%,p<0.01; **Figure 3A**). The results of subgroup analysis based on different drugs showed significant differences between the combined effect values (χ^2^ = 19.80;random effect; p <0.01; **Figure 3A**) with the RTX group(OR:0.68;95%CI=0.46-0.89,random-effect model,I^2^ = 89%,p<0.01),the TCZ group (OR:0. 95;95%CI=0.91-0.99;fixed effect model;I^2^ = 11%,p=0.34),and the TMB group(OR:0.75;95%CI=0.66-0.83;fixed effect model;I^2^ = 0%;p=0.60).The source of heterogeneity was further analysed in the RTX group (**Figure 3B**), the combined effect value of the RCT subgroup(OR:0. 87;95%CI=0.57-1.00;random effect model;I^2^ = 81%;p=0.02). Non-RCT subgroup (OR:0.55;95%CI=0.42-0. 68;fixed effect model;I^2^ = 36%;p=0.21).The results do not prove that different trial types (RCT and non-RCT) are the significant factors affecting heterogeneity. Furthermore, for the RTX group with one by one exclusion of literature, the results found that there is no heterogeneity excluding Salvi2015 (41) (I^2^ = 27.2%;p=0.25; **Figure 3B**). The funnel map test of the test results (p=0.1135) could be considered as no publication bias (**Figure 4A**).

#### 3.4.2 Disease inactivation rate

**Figure 5:**
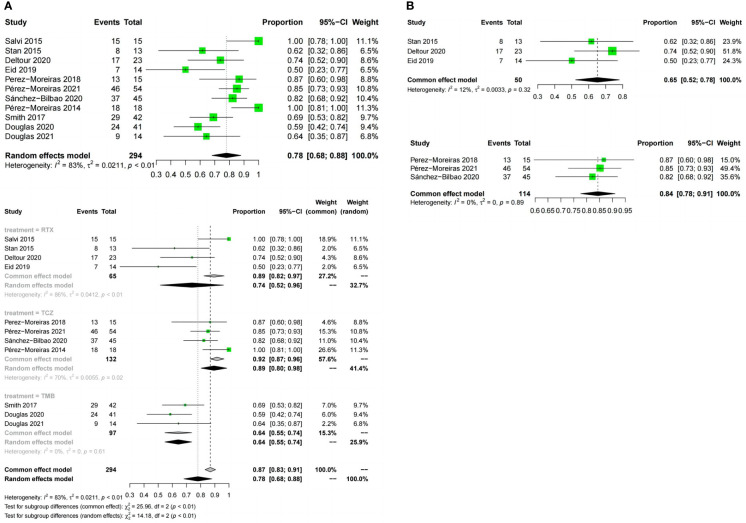
Forest plot and Funnel plots of disease inactivation rate. **(A)** Forest plot of disease inactivation rate (Combined analysis and Drugs subgroups). **(B)** Forest plot of disease inactivation rate (RTX subgroup and TCZ subgroup).

Meta-analysis concluded that 12 studies of the three drugs obtained the combined value of disease inactivation rate (OR:0.78;95%CI=0.68-0.88;random effect model;I^2^ = 83%;p<0.01;**Figure 5A**).The results of subgroup analysis based on the different drugs showed, significant differences between the combined effect values(χ^2^ = 14.18;p<0.01; **Figure 5A**), with the RTX group (OR:0.74;95%CI=0.52-0.96;random effect model;I^2^ = 86%;p<0.01),the TCZ group (OR:0.89;95% CI=0.8-0.98;random effect model;I^2^ = 70%;p<0.01),the TEP group (OR:0.64;95%CI=0.55-0.74;fixed effect model;I^2^ = 0%;p=0.61).The source of heterogeneity was further analyzed in the RTX group and the TCZ group. For the RTX group, the subgroups by test type were unchanged after one by one (I^2^ = 12%; p=0. 32; **Figure 5B**) that no heterogeneity was found; the TCZ group excluded one by one indicated that this document has a great impact on heterogeneity except Perez-Moreiras 2014 (I^2^ = 0%, p=0.89; **Figure 5B**). The funnel map test of the test results (p=0.2777) could be considered as no publication bias (**Figure 4B**).

#### 3.4.3 Reduction in CAS

**Figure 6:**
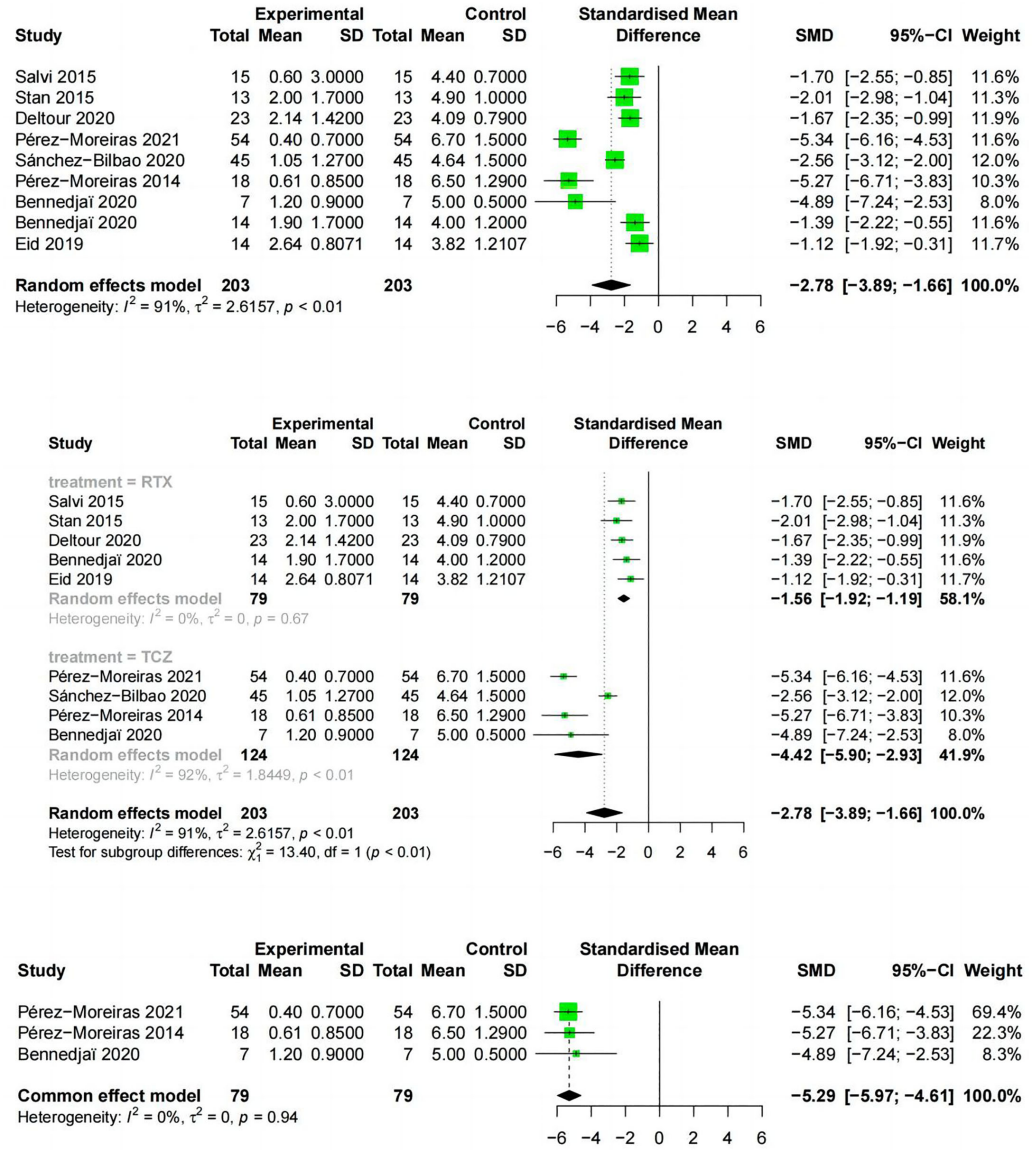
Forest plot of reduction in CAS. (Combined analysis, Drugs subgroups and TCZ subgroup).

Reduction in CAS from baseline to the end of follow-up was reported in 9 studies, including two drugs, RTX and TCZ. The literature examining the two drugs showed the CAS (SMD:-2.78;random effect model;95%CI:-3.89 to -1.66; I^2^ = 91%;p<0.01;**Figure 6**) and high heterogeneity. The results of the subgroup analysis based on RTX and TCZ showed a significant difference between the combined effect value and SMD (χ^2^ = 13.40;random effect;p<0.01; **Figure 6**) with the RTX group (SMD:-1.56;95%CI:-1.92 to -1.19;random effect model;I^2^ = 41%; p=0.16), and the TCZ group (SMD:-4.42; 95%CI:-5.9 to -2.93;random effect model; I^2^ = 92%; p <0.01). Exclusion analysis one by one indicated that this literature Sanchez-Bilbao 2020 (35) had a significant impact on heterogeneity (I^2^ = 0%, p=0.94; **Figure 6**).

#### 3.4.4 Reduction in proptosis

**Figure 7:**
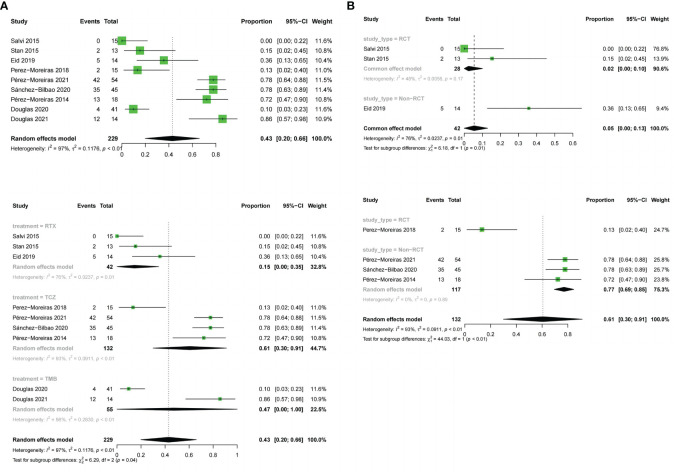
Forest plot and Funnel plots of reduction in proptosis. **(A)** Forest plot of reduction in proptosis (Combined analysis and Drugs subgroups). **(B)** Forest plot of reduction in proptosis (RTX subgroup and TCZ subgroup).

9 studies described the reduction of proptosis. The three drugs were analyzed together (OR:0.43;random effect model;95%CI:0.2 to 0.66; I^2^ = 97%; p<0.01; **Figure 7A**) and there was high heterogeneity. There was no significant difference between the combined effect scores of the three drugs in the subgroup analysis based on the three drugs (χ^2^ = 6.29; random effect; p=0.04; **Figure 7A**), with the RTX group(OR:0.15;random effect model;95%CI:0 to 0.35; I^2^ = 76%; p<0.01), the TCZ group (OR:0.61;random effect model;95%CI:0.3 to 0.91;I^2^ = 93%;p<0.01), the TEP group (OR:0.47; random effect model;95% CI:0 to 1.00;I^2^ = 98%, p<0.01). Sources of heterogeneity in the different drug groups were discussed. The RTX group, divided according to different types of trials, showed significant differences between the RCT and non-RCT combinations (χ^2^ = 6.18;fixed effect;p=0.01; **Figure 7B**), the RCT group (OR:0.02;fixed effect model;95% CI:0 to 0.1;I^2^ = 48%;p=0.17), and the overall heterogeneity (I^2^ = 76%, p=0.01).The TCZ group (χ^2^ = 44.03, stochastic effect, p<0.01; **Figure 7B**), the RCT group (OR:0.13;95%CI:0.02 to 0.4), the non-RCT group (OR:0. 77; random effect model;95%CI:0.69 to 0.85; I^2^ = 0%; p=0.89), and the overall heterogeneity (I^2^ = 93%, p<0.01). Grouping factors could be considered as significant factors leading to heterogeneity.

#### 3.4.5 Improvement in diplopia

**Figure 8:**
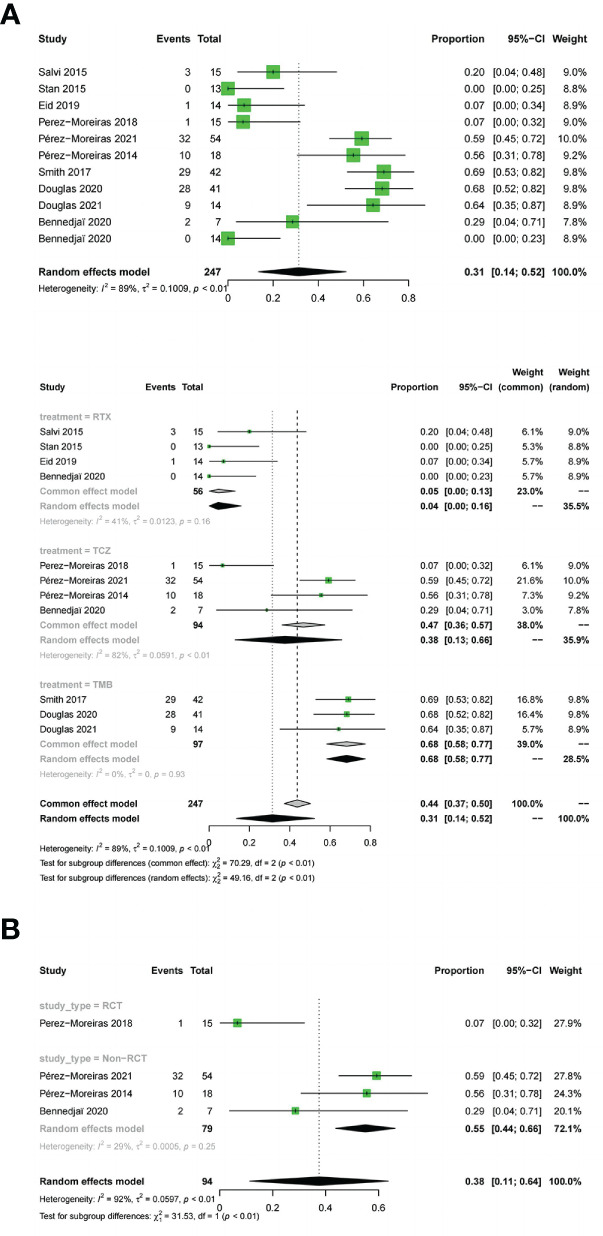
Forest plot and Funnel plots of improvement in diplopia. Forest plot of improvement in diplopia (Combined analysis, Drugs subgroups and TCZ subgroup).

11 studies described the improvement of diplopia.The three drugs (OR:0.31;random effect model;95%CI=0.14 to 0.52;I^2^ = 89%; p<0.01; **Figure 8**). The results of the subgroup analysis showed a significant difference between the combined effect values (χ^2^ = 49.16;random effect; p<0.01; **Figure 8**),and the RTX group (OR:0.05;fixed effect model;95%CI:0 to 0.13;I^2^ = 41%; p=0.16), the TCZ group (OR:0.38;random effect model;95%CI=0.13 to 0.66;I^2^ = 82%;p<0.01), the TMB group (OR:0.68;fixed effect model;95%CI=0.58 to 0.77;I^2^ = 0%;p=0.93).Sources of heterogeneity in the TCZ group were discussed.The results showed that the RCT group and non-RCT combinations (χ^2^ = 31.53;stochastic effect;p<0.01; **Figure 8**), with the RCT group (OR:0.07;95%CI:0 to 0.32), the non-RCT group (OR:0.55;random effect model;95%CI:0.44 to 0.66;I^2^ = 29%;p=0.25), the overall heterogeneity (I^2^ = 92%, p<0.01). Therefore, the grouping factor could be considered as a significant factor leading to the heterogeneity.The result of the funnel plot asymmetry test could be considered without publication bias(t =-2.09;df =7;p=0.0754; **Figure 4C**).

### 3.5 Tolerability

**Figure 9:**
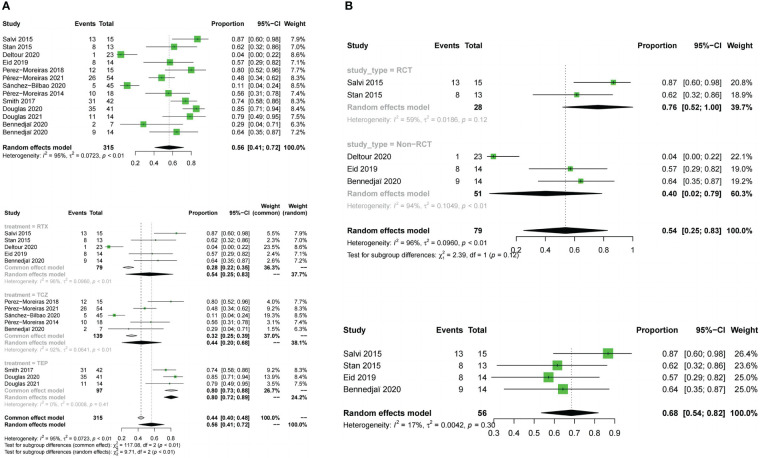
Forest plot and Funnel plots of adverse events rate. **(A)** Forest plot of adverse events rate (Combined analysis and Drugs subgroups). **(B)** Forest plot of adverse events rate (RTX subgroup).

Adverse events were evaluated in all studies. The three drugs of adverse event incidence (OR:0.56;random effect model;95%CI:0.41 to 0.72;I^2^ = 95%;p <0.01; **Figure 9A**), and with high heterogeneity. Subgroup analysis showed a significant difference between the combined effect values of the three drugs (χ^2^ = 9.71;random effect;p<0.01; **Figure 9A**), with the RTX group(OR:0.54;random effect model;95%CI:0.25 to 0.83;I^2^ = 96%;p<0.01),the TCZ group (OR:0.44;random effect model;95%CI:0.2 to 0.68;I^2^ = 92%;p<0.01), the TMB group (OR:0.8;fixed effect model;95%CI:0.723 to 0.88;I^2^ = 0%;p=0.41).Sources of heterogeneity of the RTX and TCZ groups were further discussed as follows: the RTX group, CT and non-RCT groups were not significantly different (χ^2^ = 2.39, random effect, p <0.12; **Figure 9B**). No heterogeneity was found after excluding the Deltour 2020 study (31). (I^2^ = 17%, p=0.3; **Figure 9B**). The assessment of heterogeneity in the TCZ group needs further discussion from the perspective of clinical inclusion criteria. The result of the funnel plot asymmetry test could be considered without publication bias (t =0.29;df =11;p=0.7786; **Figure 4D**).

## 4 Discussion

Intravenous GCs treatment is still the preferred method for active moderate to severe GO, and published studies have extensively reported the effects on improving clinical activity and markers of graphene oxide severity (42, 43). Recent EUGOGO recommendations include a cumulative dose of 4.5-5 g for most patients with moderate to severe active GO and a recommended dose of 7.5 g for patients with more severe or persistent/unstable diplopia (19). However, recurrence after treatment was been reported in about 20% and compressive optic neuropathy in 7.5%.This suggests that the primary role of GCs is anti-inflammatory rather than pathogenic (44). Furthermorehe efficacy of GCs treatment is restricted to moderate-to-severe GO because higher doses are associated with a higher incidence of adverse effects,and there have been reports of acute, fatal hepatotoxicity in patients treated with high doses of Intravenous GCs (45). Therefore, novel pharmacotherapy is of vital importance to improve the physical health and quality of life of patients with GO.After the initial attempt at biological therapies for GO (46), one of the most prominent types of new drug treatment for GO more recently has been monoclonal antibodies, mainly including RTX (which acts against CD20), TCZ (which acts against the IL-6 receptor) and TMB (a human monoclonal anti-IGF-1R blocking antibody), which have shown promising results in reducing symptoms and signs in moderate to severe patients. Our review also found that these three monoclonal antibodies had reliable clinical trial data from literature searches. Comparison of new therapeutic approaches is essential and requires a better understanding of each drug’s place in the treatment armamentarium for TED. To our knowledge, this is the first analysis of the efficacy of monoclonal antibodies.

**Figure 10:**
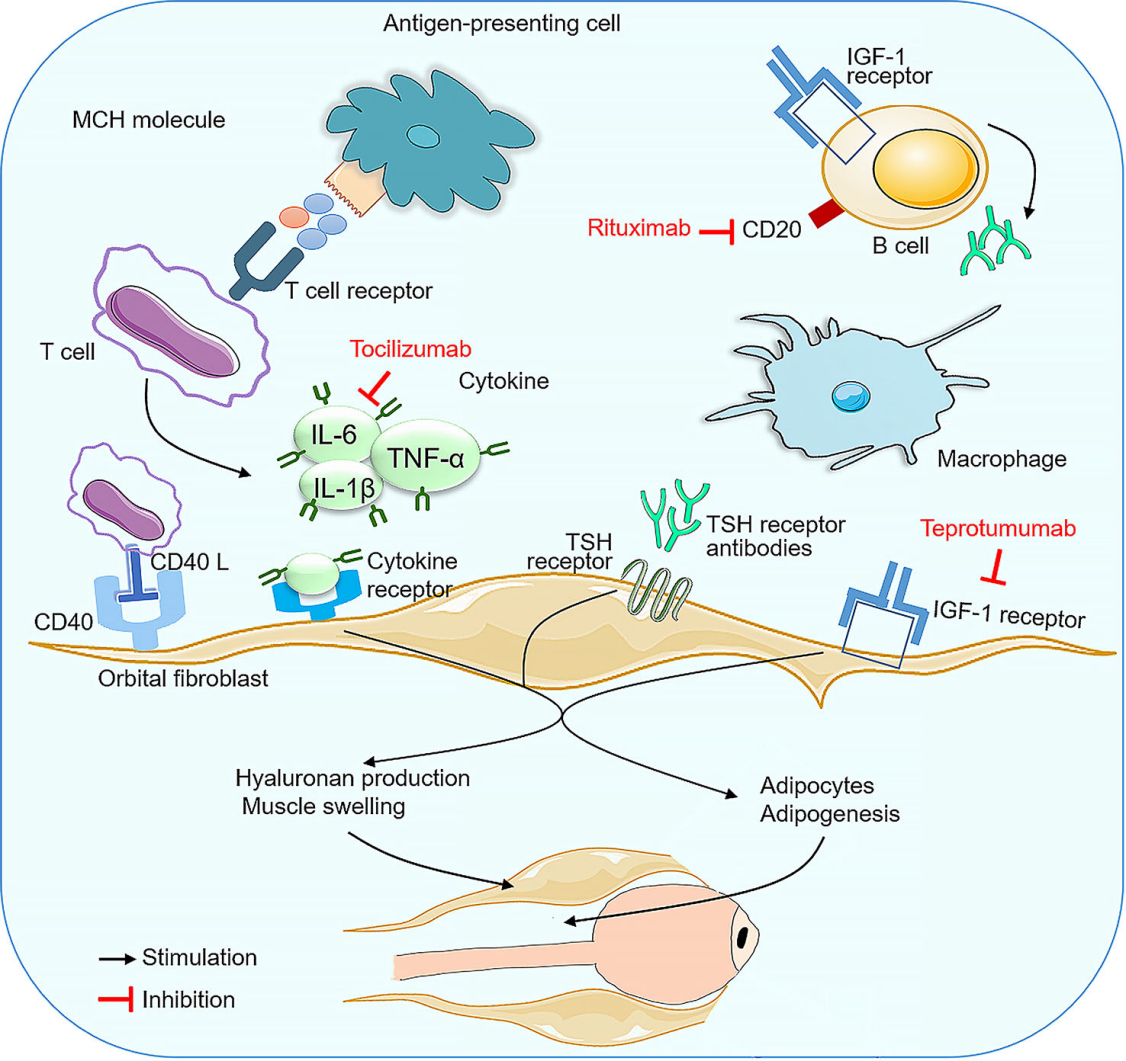
Immunopathogenesis of Graves’ophthalmopathy and therapeutic targets of mAbs.

This review compared different monoclonal antibodies for the treatment of GO, including the most recent trials. In this meta-analysis, we reviewed five RCTs and seven observational studies comparing RTX, TCZ and TMB as monotherapies for patients with active and moderate-to-severe GO, and provided a hierarchy of both efficacy and tolerability for GO interventions. Although the precise pathogenesis of GO continues to be investigated, the current evidence clearly indicates that autoimmune mechanisms clearly play an important role (**Figure 10**). Activation of B lymphocytes induces the secretion of numerous cytokines, including IFN-γ, TNF-α,IL-1 and IL-6,etc.The release of cytokines induces the synthesis and release of large amounts of glycosaminoglycans such as hyaluronan by orbital fibroblasts, causing swelling of the orbital tissues and extraocular muscles. Humoral immunity brings anti-TSH receptor antibodies into play. TSH receptor expression on orbital fibroblast membranes is increased, and binding to IGF-1 also stimulates hyaluronic acid and lipogenesis during the active phase of the disease (47).

RTX is a monoclonal antibody that targets the CD20 protein present on pre-B cells through to mature and memory B cells (48). and depletes the B cell population, leading to a reduction in the ability of B cells to present antigens, thereby reducing T cell activation and halting ongoing inflammation (49, 50). The US Food and Drug Administration (FDA) has approved RTX for the treatment of RA, Wegener’s granulomatosis, non-Hodgkin’s lymphoma and chronic lymphocytic leukaemia,as well as off-label use in other autoimmune diseases (51), and has been suggested for the treatment of GO (52). TCZ is a humanised monoclonal antibody directed against IL-6 soluble and membrane receptors, and is approved for rheumatoid arthritis (53), Castleman’s disease and systemic juvenile idiopathic arthritis (54). IL-6 is a pro-inflammatory cytokine secreted by T cells and macrophages to stimulate the immune response. It has been reported that IL-6 and its soluble receptor are activated, and high serum IL-6 receptor levels have been found in patients with active GO (55). IL-6 is also involved in the synthesis of GAGs in orbital fibroblasts and is also involved in the increase in surface TSH of these cells, and orbital volume has also been shown to be proportional to IL-6 mRNA expression levels (56–58). TMB is a recombinant, fully human, anti-IGF-1R monoclonal antibody, the first immunomodulatory agent approved by the FDA for the treatment of GO.The IGF-1R was found to be over-expressed in orbital connective tissue, T and B cells in GO patients, which produce autoantibodies capable of binding to the IGF-1R and initiating signalling from the TSHR/IGF-1R physical and functional protein complex.Autoimmune activation of orbital fibroblasts by autoantibodies with receptor agonist properties triggers and drives active (inflammatory) GO, which stimulates the release of chemoattractant cytokines, leading to fibroblast proliferation and differentiation, extracellular matrix increase, tissue expansion, oedema and extensive orbital tissue remodelling (41, 59–63). Therefore, using mAbs against IGF-1R can attenuate signaling from either TSHR or IGF-1R (64). TMB binds with high affinity to IGF1R as a pharmacological, functional inhibitor *via* its endogenous ligands (IGF1 and IGF2), blocking IGF1R activation and leading to receptor internalisation.An *in vitro* study demonstrated its efficacy in reducing fibrocyte expression of IGF-1R and TSH-R and their downstream signals, thereby blocking the induction of pro-inflammatory cytokines (65, 66).

CAS significantly predicts response to anti-inflammatory therapies, and the efficacy response rate is the main basis for determining efficacy.In our study, as monotherapy for moderate-to-severe active GO,TCZ, TMB and RTX showed significant efficacy in the main analysis.Meanwhile, we compared meanwhile three indicators, mainly based on CAS, including efficacy response rate, disease inactivation rate and CAS. According to the results of the indirect contrast, TCZ was most likely to become the best regimen based on response, followed by TMB and RTX. TCZ was also most likely to become the best treatment for reducing proptosis, followed by TMB and RTX.TMB was most likely to become the best treatment for improving diplopia, followed by TCZ and RTX.TCZ was most likely to become the best treatment for safety, followed by RTX and TMB.

The promising results of RTX in GO were first reported in 2006 (25), and since then several studies have shown significant improvement in CAS with low relapse rates after RTX infusion (67, 68). However, in 2015, two RCTs of 25 and 32 patients with GO showed conflicting results, raising concerns about the benefit of RTX in GO (26, 30, 32, 69, 70). More recently, a multi-centre retrospective study of 40 GO patients and another study of 14 patients with active and moderate to severe GO investigating the efficacy of RTX have shown that CAS is significantly improved and GO inactivation is remarkably observed, especially in the early phase of the disease. In addition, its effect on proptosis is inconsistent and has been refuted by many studies (26, 30), which is consistent with our findings that RTX was the least effective in improving proptosis. A double-blind, randomised controlled trial in Spain investigated the effects of intravenous TCZ in moderate-to-severe corticosteroid-resistant GO (33). Significant reductions in CAS and exophthalmos were observed in patients treated with TCZ after 16 weeks of treatment, and no effect on diplopia was observed. TCZ was evaluated in a prospective, non-randomised study in 18 patients with GO (previously resistant to CS) and showed a statistically significant reduction in CAS, improvement in proptosis, improvement in extraocular motility, but resolution of diplopia (71). A prospective, randomised, open-label study in patients with bilateral active steroid-resistant GO (33 TCZ group) showed the same results. Diplopia improvement remained the worst, which is consistent with our analysis that TCZ was not optimal for diplopia improvement (64, 72–78). The first multicentre, double-masked, randomised, placebo-controlled trial enrolled 88 patients, and a subsequent phase 3 trial enrolled 83 patients to investigate the efficacy of TMB in patients with active moderate to severe GO (37). The majority of patients in the TMB group had a response at week 24, resulting in better outcomes than placebo in terms of CAS,diplopia, proptosis and quality of life, and non-responders were included in an open-label extension. The plus extension study showed that the majority of patients who responded with improvement in proptosis and diplopia at week 24 continued to respond (79). The potential benefit of treatment with TMB is based on its beneficial effects on all of the symptoms of GO, especially in cases of diplopia and exophthalmos,which is consistent with our analysis. Few controlled trials of mAbs and GCs have been reported. In a 32-patient, double-blind, randomized trial of RTX vs intravenous methylprednisolone (IVMP), 100% of patients in the RTX group improved at 24 weeks compared with 69% in the IVMP group. At 52 weeks, TX was better than IVMP (in clinical activity score, lid aperture, proptosis, and diplopia score), and there were no relapses in the RTX group compared to 31% in the IVMP group. The study showed that RTX was superior to IVMP in terms of efficacy, durability and control of recurrence (30). More recently, a match-adjusted indirect comparison of TMB vs IVMP vs placebo including 12 trials by Raymond S et al (80). This meta-analysis suggested that TMB was favored over IVMP (odds ratio,2.32; 95% CI,1.07-5.03) in odds of proptosis (95% CI,-3.45 to -1.17 mm) and diplopia response (odds ratio,2.32; 95% CI,1.07-5.03),and may be twice as likely to achieve a 1 grade or higher reduction in diplopia.

However, safety is a necessary consideration in determining whether a drug is suitable for clinical use. In our included trials, the majority of patients receiving their first RTX infusion experienced mild adverse events, including mild fever, nasal congestion, infusion reaction, throat itching, and nausea. Slowing down the RTX infusion or giving intravenous hydrocortisone resolved these symptoms spontaneously (32, 81). TCZ adverse events that occurred during the trial were mainly pulmonary, gastrointestinal and renal infections, similar to those reported in rheumatoid arthritis trials (82, 83). However, there is a risk of serious adverse events exists, such as opportunistic infections, which have been reported during treatment and therefore monitoring for these during treatment is essential (33, 82, 84). Nevertheless, TCZ remains an interesting treatment option and is currently used as a second-line treatment in France.TMB was relatively poorly tolerated, with 11.9% of patients receiving it withdraw from the trials in our review due to adverse events.The adverse events were mild to moderate in severity, with hyperglycemia being the most common, which can easily attributed to therapy adjustments. Other AEs included hearing abnormalities, diarrhea, muscle cramps, alopecia and dysgeusia (31, 85). TMB has been approved by the US FDA for the treatment of GO and is currently used in clinical practice in North America (79).

Unlike previous trials, we compared different monoclonal antibodies for GO patients.The outcome measured was not just response rate, multiple databases and websites were searched for publication bias prevention.To detect potential bias, we also used funnel plots, and fortunately there was little evidence of bias.Howeverur current meta-analysis had its own limitations. Firstly, we included observational studies in addition to randomised controlled trials, which is a particular problem for monoclonal antibodies for GO, because there are very few RCTs. This issue has not been sufficiently studied due to the limited number of trials available to us.

## 5 Conclusion

In the absence of RCTs, which are the best source of evidence on the balance of risks and benefits of different treatments. These comparative efficacy results may be useful as a basis for clinical decisions and future studies. In addition, the optimal dose and potential mechanism of action of monoclonal antibodies remains to be established. There appears to be a long way to go to better understand the biological mechanism of RTX and to develop a rational therapeutic regimen. If these results are confirmed, it is likely that the treatment paradigm will change in the future. Overall, there are many encouraging advances in the treatment of Graves’ ophthalmopathy that make the future more promising for patients with this disease.

## Data availability statement

The original contributions presented in the study are included in the article/supplementary material. Further inquiries can be directed to the corresponding author.

## Author contributions

YH designed the research process. YH and JHC searched the database for corresponding articles, extracted the data, performed the statistical analyses, and wrote the manuscript with support from other. KL and XJY performed the supervision and project administration. All authors contributed to the article and approved the submitted version.
